# Are Austrian practitioners ready to use medical apps? Results of a validation study

**DOI:** 10.1186/s12911-019-0811-2

**Published:** 2019-04-24

**Authors:** Fanni Hofer, Daniela Haluza

**Affiliations:** Center for Public Health, Department of Environmental Health, Medical University of Vienna, Kinderspitalgasse 15, 1090 Vienna, Austria

**Keywords:** mHealth, Telemedicine, Principal components analysis, Healthcare personnel, Health communication, Medical technologies

## Abstract

**Background:**

As part of the mobile revolution, smartphone-based applications (apps) have become almost indispensable in today’s information society. Consequently, the use of medical apps among healthcare professionals has increased heavily over the past years. As little is known on medical app use in day-to-day clinical practice in Austria, the present study aims at closing this knowledge gap by assessing respective prevalence, readiness, and concerns among Austrian practitioners.

**Methods:**

We conducted a cross-sectional online survey among a sample of 151 Austrian doctors (mean age 45.0, SD 12.0, 55.0% females). We developed a German study questionnaire on the basis of the Practitioner Telehealth Readiness Assessment Tool (PRAT) to assess medical app use-related readiness and attitudes, and validated it using principal component analysis.

**Results:**

In our study, 74% of participants used medical apps on a daily basis, with clarity, ease of use, speed, and support in clinical routine mentioned as most important app features. However, the majority of participants perceived certain barriers to use medical apps. Younger participants used more medical apps, were more likely to use them during work, and yielded higher readiness scores. The most often used medical apps were Diagnosia and Embryotox (both 28.5%).

**Conclusions:**

Nowadays, medical apps serve as an important source of information for many doctors and are especially popular among younger physicians. The omnipresence of smartphones in the smocks of healthcare workers raised awareness for potential shortcomings regarding disruption of traditional face-to-face doctor-patient interaction among all healthcare stakeholders. This study’s finding thus highlight the need for initiating a public discussion on legal and social frameworks to successfully integrate mobile apps into everyday clinical.

## Background

The omnipresence of mobile digital devices is perceived as a characteristic feature of today’s information society. Junior doctors and also patients belonging to a new generation of “digital natives” currently enter the healthcare system [1–4]. As a consequence, international studies observe high and constantly growing rates of mobile digital device use for medical purposes [5–7]. Earlier studies have already shown that mobile phones are superior to traditional pagers as direct communication is time-saving and enhances accuracy and satisfaction with communication among hospital staff [8, 9]. The most popular types of mobile phones are smartphones such as iPhones and Android phones [10]. Abundant smartphone-based medical applications (apps) for general healthcare demands, disease diagnosis, drug reference, medical training, clinical score calculators, and literature search have been developed so far, with their number increasing day by day [11]. In addition to their obvious use in clinical communication, smartphones equipped with these apps are useful tools for practicing evidence-based medicine at point of care through rapid access to online or offline medical content [12]. These apps enable the doctor to proactively search for state-of-the-art knowledge on initial diagnosis and diagnostic refinement, drug effects and interactions [13] [14–16], laboratory reference values [17, 18], and for arithmetic operations [12, 19].

Literature suggests that the long-term effects of mobile device use by the doctor, e.g. to consult medical apps, during medical counseling on patient satisfaction and treatment efficacy are unknown yet [6, 7]. Thus, generally accepted standards are still missing to ensure smooth integration of medical apps into everyday doctor-patient communication [20, 21]. Nevertheless, medical apps can facilitate decision-making in clinical contexts, improve workflows, and reduce medical error rates, which are reasons for the constantly increasing popularity among healthcare providers worldwide [11, 22, 23].

In a German study published in 2014, the majority of doctors (82%) already owned mobile devices [6]. A US study published in 2015 found a high prevalence of medical app use of 92% among healthcare professionals [7]. Despite their abundant use, little is known on prevailing medical apps use and associated concerns of smartphones app use in medical contexts in Austria [24]. According to previous research, technology acceptance among Austrian doctors was rather low compared to other healthcare stakeholders [25–31]. The present study aimed at obtaining a comprehensive picture of the readiness to use medical apps from the perspective of Austrian practitioners, closing the current knowledge gap in this emerging field affecting medical communication, education, and healthcare delivery. For this purpose, we developed a novel German research tool on the basis of the Practitioner Telehealth Readiness Assessment Tool (PRAT), which is originally in English, and validated it using principal components analysis (PCA) [32, 33].

## Methods

### Survey tool development

We conducted a cross-sectional online survey among a non-probability purposive sample of medical doctors, thus following the Checklist for Reporting Results of Internet E-Surveys (CHERRIES) [34]. On the basis of the English version of the PRAT, we developed a German study questionnaire [32, 33]. PRAT is easily adaptable to capture the multidimensional concept of telehealth readiness in different contexts and research settings. Due to its wide distribution and high adaptability for various research purposes, we aimed at translating and validating the PRAT in German as recommended by guidelines [35–37]. In our sample, we were interested in medical app use specifically and thus modified the original English PRAT accordingly. Basically, we replaced the concept of telehealth with medical app use, where appropriate, as done in similar studies adapting PRAT [28, 30, 38]. In two forward-backward translation steps, we translated the instrument into a preliminary German survey tool, which we pre-tested in two phases for face or content validity. In the first phase (respondent debriefing, *n* ≥ 10), people without medical background (e.g. students from other faculties) were randomly addressed to evaluate the questionnaire for comprehensibility and give detailed feedback; responses of twelve voluntary participants were evaluated. After according adaptations, physicians as representatives of the target group were invited to pre-test the questionnaire in the second phase of the pretest (expert evaluation, *n* ≥ 20); responses of 23 voluntary participants were evaluated. The final survey tool was then back-translated into English for publication. The German survey tool can be obtained from the authors upon request.

### Study questionnaire

The online questionnaire collected self-reported data on socio-demographic characteristics including age (in years), gender, nationality, and region (East Austria including the capital Vienna vs. rest of Austria). We also assessed professional role (general practitioner working in private practice, specialist physician working in hospital, and specialist physician working in private practice), length of service in the three categories up to 10 years, 10–20 years, and more than 20 years, and statutory health insurance physician (SHIP, yes vs. no) to differentiate between private and panel doctors [39].

We invited participants to indicate perceived importance of features when using a medical app (important vs. unimportant). We further asked frequency of medical app use during every-day clinical routine (single choice), distinguishing frequent app users (several times a day, once a day, several times a week), rare users (once a week, less than once a week, very rare), and non-users (never). We further provided commonly used medical apps detected in the pre-test and after literature review as a drop down list and also collected eventually missing apps in a free text box. These identified choices included Diagnosia, the most widely used medical app in Austria, which is used continuously by over 5500 doctors [13], Embryotox [14], Antibiotika Thalhammer [15], and Arzneimittel Pocket [16], for laboratory reference values, i.e. Laborwerte [17], Labormedizin Pocket [18], and for arithmetic operations, i.e. MedCalc [19].

The main part of the questionnaire consisted of 17 PRAT items (as shown in Table 2) related to the three readiness domains for medical app use: A) core readiness assessing dissatisfaction with the status quo, expectation of change, and a need for medical apps, B) engagement readiness assessing awareness and perceived benefits and barriers of medical apps, and C) structural readiness assessing the development of adequate technical infrastructure and soft skills for medical app use [40].

Responses to the three categories translated in readiness level scores for medical app use for the single categories A, B, and C, and also in a global score, with higher averaged scores meaning greater readiness [41]. The global score was interpreted as follows: above 80: practitioners are in a good position to use medical apps; 60–80: certain items may adversely impact the use of medical apps; and below 60: there are barriers to successful practitioner use of medical apps.

A free text comment box at the end of the survey collected additional opinions on medical app use.

### Data collection

The online study was open and accessible to respondents from 30 January to 10 March 2018 using the complimentary web-based survey tool SoSci Survey [42]. Austrian German-speaking doctors were eligible to participate in the survey. We distributed the link to the online survey via the newsletter of the national Doctor’s Chamber, which was sent out every two weeks to members with an active newsletter subscription. The survey included a cover page providing information about the study aim. Due to the web-based nature of data collection, study subjects gave their implicit consent for participation when starting the online survey. Participation was anonymous as participants did not provide personal data and on a voluntary basis without a remuneration component. An electronic cookie that prevented multiple submissions from the same electronic device ensured single participation. The survey automatically ended for those who indicated at the start that they are not medical professionals. Respondents had the possibility to review and change their answers using a back button. We did not randomize items. Collected data were retrieved from the online survey platform and stored securely. Only members of the research team had user identification and password protected data access. We checked data for completeness and analyzed only complete questionnaires. The link to the questionnaire was opened 1104 times by unique visitors, 203 persons started the survey and 151 fully completed it (completion rate: 74.4%). Average completion time of the survey was 7.2 min (SD 1.8).

### Statistical data analysis

We conducted all statistical analyses using SPSS Version 25.0 (SPSS Inc., Chicago, IL, USA). We descriptively summarized the survey data and presented categorical data as absolute and relative frequencies and continuous data as mean and standard deviation (SD), where appropriate. We yielded a dichotomized variable for age by using median splitting to divide the sample in a younger share being 44 years and younger and an older one being older than 44 years. We determined differences between the dichotomized subgroups age (young vs. older), gender (male vs. female), and SHIP (yes vs. no) using chi^2^-tests for percentages and Mann-Whitney U tests for means. We used Cronbach’s alpha (α) to determine internal consistency of the scales. Further, as suggested by Nayar et al., we employed PCA with varimax rotation to analyze the factor structure of the 17 items of our German PRAT survey tool showing a good internal consistency of α =0.861 [38]. Our data met the assumptions for this procedure, as the Kaiser-Meyer-Olkin test of sampling adequacy of 0.866 indicated an acceptable sample size, and the Bartlett’s test of sphericity with *p* < 0.001 indicated that there are correlations in our data that are appropriate for factor analysis [43]. Further, we analyzed qualitative content collected as responses to the free text question asking for additional opinions on medical app use [44].

## Results

Average age of participants was 45.0 years (SD 12.0, range 24 to 71 years), 55.0% were females, and 50.3% belonged to the younger age group. Most respondents had more than 20 years of professional experience (41.1%) compared to those with up to 10 years and 10–20 years (31.8 and 27.2%, respectively) and were SHIPs (72.8%). Whereas only one fourth of participants (25.2%) worked as specialist physicians in private practices, the majority were general practitioners in private practices and specialist physicians working in hospitals (38.4 and 36.4%, respectively). We collected data from participants across Austria, with fewer participants from the Eastern part of Austria, which includes the capital Vienna and a high density of physicians, compared to the rest of the country (44.3 vs. 55.6%).

We found that about half of our participants were frequent app users (51.7%), 22.5% were rare and 25.8% were non-users. The two top ranked features of medical apps referred to user-friendliness with 98.7% of participants perceiving that clarity and ease of use as most important. Support in clinical routine (95.4%), speed (94.0%), and proof of quality (89.4%) were also major reasons for using these apps. Aspects like free use (62.9%), electronic use of health-related information (50.3%), use of health data via app (41.7%), feedback from others (27.2%), and use by a colleague (18.5%) were perceived as subordinate compared to the aforementioned features.The most often used medical apps were Diagnosia and Embryotox (both 28.5%). Participants were less familiar with other medical apps such as Antibiotika (Thalhammer) (19.9%), MedCalc (16.6%), Laborwerte (13.2%), Arzneimittel Pocket and Labormedizin Pocket (both: 9.3%).

Average amount of used medical apps was 2.4 (SD 1.7, range 1–9) with the majority of participants indicating to use one (40.4%) or two apps (23.2%). Although we did not find statistically significant subgroup differences for gender and SHIP, average amount of medical apps used was higher among younger participants compared to older ones (mean 2.8, SD 1.7 vs. mean 2.0, SD 1.6, *p* < 0.001). Whereas 27.6% of younger participants reported on using one app, 53.3% of the older ones did so, and whereas 5.3% of the older participants reported on using four medical apps, 18.4% of the younger did so.

More than half of participants agreed that the use of medical apps during the medical consultation disturbs the relationship with the patient (59.6%), 39.1% used medical apps in everyday medical practice, and only 18.5% agreed that they would miss using medical apps (Table 1). Nearly half of participants agreed on offering their patients to contact them by phone (49.7%) and via online communication tools (47.0%). We did not find statistically significant differences for gender, but we observed differences for age group and SHIP. Younger participants were less likely than older ones to offer their patients the opportunity to contact them by phone (35.5% vs. 64.0%, *p* < 0.001) as well as by online communication (38.2% vs. 56.0%, *p* < 0.05), and they were more likely to use medical apps during medical work (52.6% vs. 35.3%, *p* < 0.05). Participants with SHIP status were more likely to use medical apps during work (26.8% vs. no 43.6%, *p* < 0.001) and to give their patients the opportunity to contact them by phone (75.6% vs. no 40.0%, %, *p* < 0.05) and via online communication tools (68.3% vs. no 39.1%, *p* < 0.05).

**Table 1:** Views on medical app use

Items	Do not agree		Neutral		Agree		Mean	SD
	n	%	n	%	n	%		
In my opinion, the use of medical apps during the medical consultation disturbs the relationship with the patient.	26	17.2	35	23.2	90	59.6	2.42	0.77
I give patients the opportunity to contact me by phone.	46	30.5	30	19.9	75	49.7	2.19	0.88
I give patients the opportunity to contact me via Internet-based means of communication.	53	35.1	27	17.9	71	47.0	2.12	0.90
I do not use medical apps in everyday medical practice.	59	39.1	23	15.2	69	45.7	2.07	0.92
I could not imagine everyday medical practice without the use of medical apps.	91	60.3	32	21.2	28	18.5	1.58	0.79

Table 2 shows participants` readiness assessment for medical app use. We found the highest average total ratings for engagement readiness (mean 3.4, SD 0.8) compared to core readiness (mean 3.2, SD 0.7) and structural readiness (mean 3.0, SD 0.7). We did not find statistically significant subgroup differences for SHIP, but we observed differences for age in regard to engagement readiness with higher average ratings for younger participants compared to older ones (mean 3.5, SD 0.8 vs. mean 3.3, SD 0.8) and gender for core readiness with higher average ratings for females compared to males (mean 3.3, SD 0.6 vs. mean 3.1, SD 0.8, both: *p* < 0.05). In the core readiness category, the item “I have firsthand experience of the negative effects of isolation from healthcare services.” yielded highest agreement levels (mean 3.6, SD 1.1). As for engagement readiness, the item “I have the need to interact with other practitioners.” was rated highest compared to the other items (mean 3.9, SD 0.7). In the category structural readiness, participants expressed highest agreement with the item “I have dealt with apprehensions about the reliability of medical apps and have good technical support and backup plans.” (mean 3.5, SD 1.2).

**Table 2:** Readiness assessment for medical app use

Categories for readiness assessment for medical app use		
	Mean	SD
A) Core readiness		
I have a feeling of dissatisfaction with the current available ways of delivering care, e.g. status quo.	2.81	1.12
I have firsthand experience of the negative effects of isolation from healthcare services (professional and educational).	3.61	1.12
I have a driving need to address a public or patient healthcare problem (as opposed to a practitioner specific one) that could be met by medical apps.	3.24	1.06
Total	3.22	0.72
B) Engagement readiness		
I am an innovator and/or champion for medical app use.	3.73	1.03
I have a sense of curiosity about the influences of medical app use on improving the delivery of health care (potential benefits).	3.80	0.94
I have respect for others in the medical team using medical apps.	3.28	1.06
I have the need to interact with other practitioners.	3.93	0.69
I have examples and evidence of medical app use in similar contexts.	2.87	1.11
I communicate with other practitioners and the public concerning the benefits of medical app use.	2.89	1.16
I am willing to make the initial extra investment in time.	3.20	1.15
Total	3.39	0.77
C) Structural readiness		
I believe medical app use can address scheduling concerns and apprehensions about overextended workloads.	2.79	1.01
I have 24-h access to medical apps.	3.32	1.27
I have reimbursement plans for medical app in place.	2.02	1.08
I have dealt with apprehensions about the reliability of medical apps and have good technical support and backup plans.	3.49	1.19
I have access to an established reliable and available clinical consultation network (human) when using medical apps.	3.30	0.98
I am provided with reliable clinical content and continuing medical education for medical app use.	2.79	1.11
I attend to issues regarding liability and licensing when using medical apps.	2.85	1.18
Total	2.94	0.67

Table 3 shows average category scores and global score for readiness to use medical apps. In relation to the respective maximum achievable score, core readiness and structural readiness yielded lower scores (64.4 and 68.5%) compared to engagement readiness (79%). While the global score did not differ for gender and SHIP, we found statistically significant differences for age groups with higher scores for the younger participants compared to the older ones (mean 55.6 SD 10.2 vs. mean 52.3 SD 10.1, *p* < 0.05).

**Table 3:** Average scores for practitioner readiness to use medical apps

Readiness scores	Maximum points	Items	Mean	SD	Median	Range	% of maximum points
Core readiness	15	3	9.66	2.17	10	3–15	64.4
Engagement readiness	30	7	23.70	5.41	24	7–35	79.0
Structural readiness	30	7	20.55	4.66	21	8–35	68.5
Global score	75	17	53.91	10.23	54	25–83	71.9

Moderate to low ratings in the readiness assessment tool as reported above translated into three readiness levels showed that the majority of participants (70.9%) perceived barriers to use medical apps (low readiness level, < 60 points). Noteworthy, 28.5% of participants reached a moderate readiness level (60–80 points), whereas only one participant (0.7%) reached a high readiness level (> 80 points). Readiness levels did not differ among subgroups.

Table 4 shows results of PCA, revealing a four-factor structure of the 17-item readiness assessment tool. The first factor, Advocacy, embraced 13 items of all three readiness categories with all items from the engagement readiness domain (α = 0.895). The second factor, Skepticism, was built from two items of the core readiness domain (α = 0.502). Although the third factor, Liability, and the forth factor, Reliability, included only one item of the structural readiness category each, we did not limit the maximum number of factors to two, as this would explain only 45.6% of the variance (four factors: 60.4%).

**Table 4:** Principal component analysis: A) Core readiness, B) Engagement readiness, and C) Structural readiness

	Factors			
	1	2	3	4
1. Advocacy				
I am an innovator and/or champion for medical app use. (B)	0.83			
I have a driving need to address a public or patient healthcare problem (as opposed to a practitioner specific one) that could be met by medical apps. (A)	0.82			
I am willing to make the initial extra investment in time. (B)	0.78			
I have a sense of curiosity about the influences of medical app use on improving the delivery of health care (potential benefits). (B)	0.76			
I communicate with other practitioners and the public concerning the benefits of medical app use. (B)	0.75			
I have the need to interact with other practitioners. (B)	0.71			
I have examples and evidence of medical app use in similar contexts. (B)	0.65			
I believe medical app use can address scheduling concerns and apprehensions about overextended workloads. (C)	0.62			
I have access to an established reliable and available clinical consultation network (human) when using medical apps. (C)	0.62			
I have 24-h access to medical apps. (C)	0.61			
I have respect for others in the medical team using medical apps. (B)	0.59			
I am provided with reliable clinical content and continuing medical education for medical app use. (C)	0.48			
I have reimbursement plans for medical app in place. (C)	0.45			
2. Skepticism				
I have a feeling of dissatisfaction with the current available ways of delivering care, e.g. status quo. (A)		0.76		
I have firsthand experience of the negative effects of isolation from healthcare services (professional and educational). (A)		0.56		
3. Liability				
I attend to issues regarding liability and licensing when using medical apps. (C)			0.56	
4. Reliability				
I have dealt with apprehensions about the reliability of medical apps and have good technical support and backup plans. (C)				0.51

Only a small minority of participants shared their opinions in the free text comment box at the end of the survey (*n* = 17, 11.3%). Seven out of the 17 comments dealt with general comments regarding the survey tool, whereas ten stand-alone comments were personal view and observations and general comments on health app use.

Two participants gave reasons for barriers of mobile app use.

*“I would use many more apps if mobile phone reception was better in the General Hospital of Vienna. An offline version of an app is very important because in many hospitals reception is a problem.”*

*“The use of apps in everyday working life means that you always have to plug in the smartphone, which should be a no-go - the cell phone should not be in the smock (even if it is different in everyday life). My office employees are not allowed to use their mobile phones at work either, and that is clear to them without reservation!”*

Another participant wished that professional organizations should inform on evidence-based medical apps.

*“More info about existing apps from the Austrian Doctors Chambers incl. rating of the quality would be great!”*

Another emphasized the empowerment potential of apps in the sense of telehealth and telemonitoring.

*“Key aspect of medical apps: the patient as co-producer of his health/anamnesis.”*

Critical respective views were also raised:

*“The closer to the patient, the lonelier the decision, the more complicated and theory-weak is often scientific support.”*

*“Electronics in medicine is not only a blessing but also diabolical; DIRECT Contact with the patient is getting shorter and shorter, and we are moving more and more into a "doctor-free world.”*

Participants exemplarily described their personal ways to use medical apps in everyday clinical routine.

*“Apps are sometimes a good supplement. I am faster with tools on the laptop/PC. Here I can use several info channels at the same time.”*

*“I like the App First Aid, otherwise I research on drugs and AI via Austria Codex online.”*

*“I communicate with patients via colleagues who may speak their foreign language. Basically, you do not need apps or a mobile phone constantly to find things, but you should have a lot in your own head.”*

*“So far, the Austria Codex has sufficed me, occasionally Embryotox over the normal Internet.”*

*“I communicate with patients via WhatsApp or Facebook Messenger, but do not disclose any findings.”*

## Discussion

In the pertinent literature, rates of medical app use among healthcare staff varies considerably and ownership of a smartphone might be a proxy, but not equivalent to medical app use in general and also in front of patients [5, 6, 45]. In the present study, 74% of study subjects used medical apps in daily clinical practice and about half were frequent users. So, our Austrian sample of practitioners showed a lower medical app use compared to the use rate of over 90% among US and British healthcare professionals [7, 45].

Nowadays, an unmanageable number of these apps are commercially available and numbers will definitely rise in the near future and users would like to choose from a broad variety of easy to use and low cost medical apps [2, 5, 7]. Studies found that disease diagnosis/management, drug reference apps, and medical calculator apps were most commonly used in clinical context [5, 9]. We thus asked for most popular medical app types and found an unexpected wide range. However, study subjects did prefer using established apps basically for drug effects and interactions, for laboratory reference values, and for arithmetic operations.

We used a novel survey tool for assessing readiness to use medical apps. Results of PCA suggested that the tripartite structure as described in the original PRAT, i.e. core readiness, engagement readiness, and structural readiness, does not picture aspects of medical app use efficiently [33, 34]. Thus, using the items without the structure set by those three readiness components might be more useful to capture the readiness to use medical apps.

Reasons for low to moderate readiness levels to use medical apps as found in our sample could be manifold. In a US study, few doctors used their smartphones while with patients or during medical procedures [7]. In our survey, only 17% of participants perceived that the use of medical apps during the medical consultation would not disturb the delicate relationship with the patient. Although 39% used medical apps in everyday medical practice, only 19% could not imagine everyday medical practice without using medical apps. Also, in a German study, only about 8% of doctors avoided their use during patient contact because they felt that patients might feel uncomfortable with these devices [6, 45]. The observed halting attitude towards using medical apps during face-to-face consultation might root in the feeling that using a smartphone in front of a patient in need for medical advice and treatment is ethically and socially problematic. Potential considerations influencing smartphone use in clinical settings might relate to perceived level of relevance to patient care as well as appropriateness and expected disruptiveness of the behavior [7]. Nevertheless, examples of telehealth readiness assessment among other use cases suggest that experience in the use of novel technologies might increase the readiness to use them [46].

A qualitative analysis of the free text comments revealed barriers towards using apps such as problems with smartphone reception in hospitals and the perception that the use of apps in everyday work is a “a no-go”, as one participant put it, i.e. it might be inconsistent with good clinical practice. There might also be the fear that doctors are more and more replaced by information technology and the direct interaction with the patient is lost in a digitalized world. Clearly, the practitioner’s primary tasks are disrupted by internally or externally initiated smartphone use [47]. Thus, in public Austrian hospitals, use of private smartphones is often generally banned for employees during service time, with exceptions depending on the hospital providers` internal code of conduct. Recently, increased disturbances caused by patients and their relatives using mobile phones to document ongoing treatments led to an extension of the ban to non-employees in several Austrian healthcare facilities [48].

Smartphones allow healthcare providers as well as patients to communicate efficiently and collect medical data easily. As doctors are increasingly using medical apps to facilitate clinical decision making, smartphones play a crucial role in medical care nowadays [49]. This development causes a variety of practical, ethical, and legal concerns regarding safety and confidentiality issues when patient data are stored on privately owned mobile devices will likely lead to increased control by regulatory bodies. Storing and processing patient data was not very common in a survey among German doctors (5%), although reliability of the technical features for this specific use seems not to be doubted [6]. In synopsis with the currently available literature, we suggest that conceptual frameworks are still missing to tackle the invasion of mobile devices in the form of the omnipresent smartphone in clinical settings, not only those in the smocks of healthcare workers, i.e. medical students, nurses, and doctors, but also those in patients` possession. Future research is needed to entangle the impact of infrastructure issues such as poor wireless reception and facility policies against smartphone use on physician perceptions that the doctor-patient relationship is harmed by smartphone usage during medical interview.

This study adds to the existing literature so far unknown scientific evidence on readiness for medical app use and respective activities and opinions among Austrian physicians. However, the findings of this study are subject to several potential sources of bias that should be noted as limitations. To distribute our online survey, we used the regular newsletter of the Doctor’s Chamber to reach as many nationwide subscribers as possible. Thus, we were not able to obtain exact information of the response rate, which is in general expected to be low in a study population of healthcare providers [50]. Although the number of participants was higher or similar when compared to related studies, we could not comment on representativeness and generalizability of the study [5, 7, 28, 45, 51]. We collected self-reported information reflecting personal experience, which introduced survey response bias [52]. By using an online survey tool, we ensured time- and place-independent participation among Internet-savvy Austrian practitioners that very likely resemble the target group of medical app use. Thus, these advantages of the sampling technique outweigh potential disadvantages [53].

We developed and validated a new and so far unpublished German study questionnaire to assess medical app use readiness for an online survey. We therefore translated the original English PRAT and also modified it to collect data on medical apps rather than telehealth. PCA showed that further studies could abstain from the PRAT-predetermined structure and use items with high factor loadings only. The accordingly modified survey tool would then most likely show increased psychometric properties, with notably limited comparability to already published international findings. Further larger-scale studies could use this research tool to assess regional and national differences of technology acceptance and according trends, and also verify internal consistency and construct validity.

## Conclusions

The Austrian healthcare system is perceived as one of the best worldwide, as it provides almost fully covered high-quality care with free choice of providers and unrestricted access to general practitioners, specialist physicians, and hospitals [54]. Though, the decentralized structure of healthcare provision adjusted to local preferences not only leads to high healthcare expenditures, but also causes inadequate coordination of national telehealth strategies. Thus, we expected that especially Austrian doctors would appreciate the low threshold of using private smartphones in combination with a wide variety of available medical apps to practice medicine in a self-determined manner. However, this study found a quite low readiness to use medical apps, potentially due to the perceived lacking technology acceptance among patients and institutional bans to smartphone use during patient care.

Notably, younger participants used more medical apps, were more likely to use them during work, and yielded higher readiness scores compared to older ones. So, with a new generation of “digital natives” gradually entering the healthcare system as junior doctors and also patients, popularity of medical apps will definitely rise. Decision-makers should thus strive at providing suitable conceptual, legal, ethical, and social frameworks to smooth the integration of useful app in ever-day clinical care. A vital first step should include increasing communication and cooperation between eminent healthcare stakeholders from private and public health organizations as well as patient advocates.

## Abbreviations

PCA: Principal component analysis
PRAT: Practitioner telehealth readiness assessment tool
SD: Standard deviation
SHIP: Statutory health insurance physician
α: Cronbach’s alpha
